# Kernelized multiview signed graph learning for single-cell RNA sequencing data

**DOI:** 10.1186/s12859-023-05250-y

**Published:** 2023-04-04

**Authors:** Abdullah Karaaslanli, Satabdi Saha, Tapabrata Maiti, Selin Aviyente

**Affiliations:** 1 Department of Electrical and Computer Engineering, Michigan State University, East Lansing, MI USA; 2 Department of Biostatistics, The University of Texas MD Anderson Cancer Center, Houston, TX USA; 3 Department of Statistics and Probability, Michigan State University, East Lansing, MI USA

**Keywords:** Gene regulatory networks, Single cell, Graph signal processing, Graph learning

## Abstract

**Background:**

Characterizing the topology of gene regulatory networks (GRNs) is a fundamental problem in systems biology. The advent of single cell technologies has made it possible to construct GRNs at finer resolutions than bulk and microarray datasets. However, cellular heterogeneity and sparsity of the single cell datasets render void the application of regular Gaussian assumptions for constructing GRNs. Additionally, most GRN reconstruction approaches estimate a single network for the entire data. This could cause potential loss of information when single cell datasets are generated from multiple treatment conditions/disease states.

**Results:**

To better characterize single cell GRNs under different but related conditions, we propose the joint estimation of multiple networks using multiple signed graph learning (scMSGL). The proposed method is based on recently developed graph signal processing (GSP) based graph learning, where GRNs and gene expressions are modeled as signed graphs and graph signals, respectively. scMSGL learns multiple GRNs by optimizing the total variation of gene expressions with respect to GRNs while ensuring that the learned GRNs are similar to each other through regularization with respect to a learned signed consensus graph. We further kernelize scMSGL with the kernel selected to suit the structure of single cell data.

**Conclusions:**

scMSGL is shown to have superior performance over existing state of the art methods in GRN recovery on simulated datasets. Furthermore, scMSGL successfully identifies well-established regulators in a mouse embryonic stem cell differentiation study and a cancer clinical study of medulloblastoma.

## Background

Gene expression arises from a network of regulatory interactions between transcription factors, co-factors and signaling molecules [1, 2]. Elucidating the topology of this underlying transcriptomic network is essential for understanding the mechanisms that govern complex biological processes in human physiology and pathology. Identifying the differences in transcriptional regulation between normal and disease states helps in revealing the specific biological and biochemical pathways relevant to disease mechanisms and progression [3, 4].

A major focus area in clinical research lies in studying the changes in gene coexpression networks across different tissues, cell types/states, and conditions. For example, in the extensively studied breast cancer datasets from the cancer genome atlas, there are four main subtypes of breast cancer [5]. The variation between these subtypes holds the key to inferring how genes transcriptionally regulate each other and how their expressions and interactions change across subgroups. In addition one would expect the gene relationships corresponding to different subtypes to be similar to each other since they originate in the same tissue, but also posses crucial differences since they are in different stages of disease progression [6–8]. Thus, instead of estimating a single network for all the subtypes, constructing class-specific graphical models for different conditions will provide a more robust and deeper understanding of group-specific characteristics.

Recent advances in next generation sequencing technologies have made it possible to profile the transcriptomes of individual cells, hence capturing expressions of thousands of genes at a cellular resolution. Dozens of algorithms have been proposed for the reconstruction of gene regulatory networks from single cell RNA sequencing (scRNA-seq) datasets [9, 10]. This has further enabled novel insights into the transcriptional regulation underlying various biological processes, including cancer progression [11] and embryonic development [12]. Most of these algorithms, however estimate a single gene regulatory network, assuming the data samples to be identically and independently distributed; hence ignoring the presence of natural subgroups that may be present within the data. Given the assumption of a grouped dataset, one should be able to apply these algorithms to estimate networks from each subgroup separately; but this procedure of independent group-wise network estimation will fail to model the shared structures between the subgroups, eventually leading to information loss. Therefore, there is a pressing need to develop joint graph estimation models that would allow information borrowing across subgroups while retaining subgroup specific heterogenity.

Multiple algorithms have been proposed for joint estimation of networks from high dimensional data. Most of these methods assume that the data has a Gaussian distribution. Seminal papers by [6, 13] paved the way for penalized estimation of multiple Gaussian graphical models, and demonstrated the use of lasso based penalty functions for better estimation across multiple groups. Later, Danaher et al. [6] proposed the fused graphical lasso and the group graphical lasso penalties for better estimation. These methods however are not directly applicable to single cell datasets. Despite many advantages, scRNA-seq datasets are undermined by a series of technical limitations, such as drop-out events (expressed genes undetected by scRNA-seq) and a high level of noise, which renders void the assumption of gaussianity [14–16]. Few methods have been proposed for joint estimation of multiple networks from scRNA-seq datasets. Mukherjee et al. [17] developed PIPER, a penalized local Poisson graphical model [18] for joint estimation of multiple networks in scRNA-seq datasets. One of the main limitations of PIPER is that the Poisson distribution has one single parameter characterizing both the mean and the standard deviation. Single cell datasets would be better characterized by a negative binomial distribution which has a separate dispersion parameter or a zero inflated negative binomial distribution which could account for the excessive zeroes. To account for the non-Gaussian nature of the scRNA-seq datasets, Wu et al. [19] proposed a modification of the joint Gaussian copula graphical model based on the Gaussian copula transformation proposed in [20]. To facilitate estimation of Kendall’s $\tau$ correlation matrix in the presence of dropouts they propose a modified Kendall’s $\tau$ metric that only utilizes the completely observed values, and excludes the missing values. Dong et al. [21] proposed a three step hybrid joint estimation strategy that relies on (a) integrated application of a Bayesian zero inflated Poisson based model imputation strategy and single cell imputation technique McImpute [22, 23], (b) data Gaussianization [24] and eventually (c) joint estimation of a Gaussian graphical model [6]. Contrary to [17], the last two proposed approaches estimate graphical models for continuous data and rely on a data transformation step for making the data continuous.

Recent work in graph signal processing (GSP) extends classical signal processing concepts to data defined on nodes of a graph, i.e. *graph signals* [25]. GSP based graph learning (GL) approaches infer the graph structure from the observed graph signals based on assumptions made about the relation between the signals and the unknown graph [26]. Since graph signals are represented explicitly in the graph frequency domain, GSP based GL has more flexibility in modeling signals compared to previous network inference methods, such as statistical models reviewed above for GRN inference. Therefore, in this work, we focus on GSP based GL for the joint inference of multiple GRNs, where gene expressions from cells are considered as graph signals on the unknown GRNs. Existing GL algorithms [27–30] have two important shortcomings for multiple GRN learning. First, they cannot learn signed graphs, which is a more suitable model for GRNs as they include activating and inhibitory edges. Second, with the exception of [30], they can only learn a single graph. Thus, they are not applicable to the joint inference of multiple GRNs problem.

In this paper, we present a multiple signed graph learning algorithm (scMSGL) for joint inference of GRNs from multiple classes (conditions/disease states). Based on the method developed in [31], scMSGL learns multiple GRNs by deriving an optimization problem using three assumptions: (i) expressions of genes connected with activating edges are similar to each other, (ii) expressions of genes connected with inhibitory edges are dissimilar to each other, and (iii) GRNs corresponding to the different datasets are related to each other. Thus, scMSGL optimizes the total variation of graph signals to learn signed graphs while ensuring that the learned signed graphs are similar to each other through regularization with respect to a learned signed consensus graph. The proposed method has several advantages over existing approaches. First, it performs joint GRN inference taking advantage of the shared information across datasets while not making any specific parametric assumptions about the data. Second, during application to single cell data, scMSGL is kernelized as in [31] to take the structure of scRNA-seq data into account. For instance, it can employ proportionality measures to reflect relative rather than absolute abundance or zero-inflated Kendall’s tau to handle drop-outs [32]. Finally, the proposed method learns an additional consensus graph, which captures the common structure across all graphs.

## Methods

### Graphs

A weighted undirected graph can be denoted as $G=(V, E, {\textbf {W}})$ where *V* is the node set with $|V|=n$, *E* is the edge set and ${\textbf {W}}$ is the adjacency matrix with $W_{ij}$ the weight of the edge between nodes *i* and *j*. *G* is an unsigned graph, if edge weights are constrained to only positive values. Combinatorial Laplacian matrix of the unsigned graph *G* is ${\textbf {L}}= {\textbf {D}}- {\textbf {W}}$ where ${\textbf {D}}$ is the diagonal matrix with node degrees, i.e. $D_{ii} = \sum _{j=1}^n W_{ij}$. The Laplacian matrix is positive semi-definite, thus its eigendecomposition is ${\textbf {L}}= {\textbf {V}}\varvec{\Lambda }{\textbf {V}}^\top$ where $\varvec{\Lambda }$ is the diagonal matrix of eigenvalues. We assume the eigenvalues of ${\textbf {L}}$ are ordered such that $0 = \Lambda _{11} \le \Lambda _{22} \le \dots \le \Lambda _{nn}$.

If the edge weights are allowed to take on negative values, *G* is a signed graph. A signed graph *G* can be decomposed into two unsigned graphs, ${G}^{{+}} = (V, {E}^{{+}}, {{\textbf {W}}}^{{+}})$ and ${G}^{{-}} = (V, {E}^{{-}}, {{\textbf {W}}}^{{-}})$, where ${W}^{{+}}_{ij} = W_{ij}$ (${W}^{{-}}_{ij} = |W_{ij}|$) if $W_{ij} > 0$ ($W_{ij} < 0$), and 0, otherwise.

### Graph signals

A graph signal over an unsigned graph *G* is a function $x: V \rightarrow \mathbb {R}$ and can be represented by a vector ${\textbf {x}}\in \mathbb {R}^n$ where each $x_i$ is the signal value on node *i*. Graph Fourier transform (GFT) of ${\textbf {x}}$ can be defined using the spectrum of ${\textbf {L}}$ as its eigenvalues and eigenvectors provide a notion of frequency, i.e., small eigenvalues correspond to low frequencies and large ones to high frequencies [25, 33]. GFT of ${\textbf {x}}$ is defined as $\widehat{{\textbf {x}}} = {\textbf {V}}^\top {\textbf {x}}$ and inverse GFT is ${\textbf {x}}= {\textbf {V}}\widehat{{\textbf {x}}}$. Thus, ${\textbf {x}}$ is the linear combination of eigenvectors of ${\textbf {L}}$ with the coefficients determined by $\widehat{{\textbf {x}}}$. If most of the energy of $\widehat{{\textbf {x}}}$ is concentrated in the entries corresponding to the small eigenvalues, ${\textbf {x}}$ has a low-frequency representation in the graph Fourier domain. On the other hand, if its energy is concentrated in the entries corresponding to large eigenvalues, it has a high-frequency representation. The total variation of ${\textbf {x}}$ with respect to *G* can then be quantified as:

1 $$\begin{aligned} \textrm{tr}(\widehat{{\textbf {x}}}^\top \varvec{\Lambda }\widehat{{\textbf {x}}}) = \textrm{tr}({\textbf {x}}^\top {\textbf {V}}\varvec{\Lambda }{\textbf {V}}^T {\textbf {x}}) = \textrm{tr}({\textbf {x}}^\top {\textbf {L}}{\textbf {x}}), \end{aligned}$$

whose small(large) values indicate that ${\textbf {x}}$ has low(high) frequency representation on *G*.

For a graph signal ${\textbf {x}}$ defined on a signed graph *G*, total variation can be quantified based on the decomposition of *G* into ${G}^{{+}}$ and ${G}^{{-}}$. Namely, let ${{\textbf {L}}}^{{+}}$ and ${{\textbf {L}}}^{{-}}$ be the Laplacian matrices of ${G}^{{+}}$ and ${G}^{{-}}$, then we define two total variation values for ${\textbf {x}}$: $\textrm{tr}({\textbf {x}}^\top {{\textbf {L}}}^{{+}} {\textbf {x}})$ and $\textrm{tr}({\textbf {x}}^\top {{\textbf {L}}}^{{-}} {\textbf {x}})$.

### Single view signed graph learning

Given a set of graph signals $\{{\textbf {x}}_i \in \mathbb {R}^n\}_{i=1}^p$ that are defined on an unknown unsigned graph *G*, Dong et al. [27] proposed to learn the structure of *G* with the assumption that signals admit low-frequency representation in the graph Fourier domain of *G*. Thus, *G* can be learned by minimizing (1) with respect to ${\textbf {L}}$ as follows:

2 $$\begin{aligned} \mathop {\textrm{minimize}}\limits _{{\textbf {L}}\in \mathbb {L}}\ \textrm{tr}({{\textbf {X}}}^\top {\textbf {L}}{\textbf {X}}) + \alpha \Vert {\textbf {L}} \Vert _{F}^2\ {{\,\mathrm{\mathrm{subject\ to}}\,}}\textrm{tr}({\textbf {L}}) = 2n, \end{aligned}$$

where ${\textbf {X}}\in \mathbb {R}^{n\times p}$ is the data matrix whose columns are ${\textbf {x}}_i$’s, $\mathbb {L}=\{{\textbf {L}}: L_{ij} = L_{ji} \le 0\ \forall i\ne j,\ {\textbf {L}}\varvec{1}= \varvec{0}\}$ is the set of valid Laplacian matrices. The first term in (2) measures the total variation of the graph signals. The second term is the Frobenius norm of ${\textbf {L}}$ and controls the density of the learned graph. Finally, the last constraint is added to prevent the trivial solution ${\textbf {L}}= \varvec{0}$.

In [31], (2) is extended to learn an unknown signed graph *G* based on the assumption that the graph signals admit (i) low-frequency (smooth) representation over ${G}^{{+}}$, and (ii) high-frequency (nonsmooth) representation over ${G}^{{-}}$. Smoothness and non-smoothness of the graph signals with respect to signed graphs are defined as follows: (1) Signal values on nodes that are connected with positive edges are similar to each other; (2) Signal values on nodes that are connected with negative edges are dissimilar to each other. These assumptions imply that if genes *i* and *j* are connected by an activating edge, their gene expressions should be similar, i.e low-frequency. On the other hand, if *i* and *j* are connected by an inhibitory edge, their expressions should be dissimilar, i.e., high frequency. These assumptions are biologically reasonable and have been validated in [31]. Based on these assumptions, the signed graph *G* is learned by minimizing $\textrm{tr}({\textbf {X}}^\top {{\textbf {L}}}^{{+}} {\textbf {X}})$ with respect to ${{\textbf {L}}}^{{+}}$ and maximizing $\textrm{tr}({\textbf {X}}^\top {{\textbf {L}}}^{{-}} {\textbf {X}})$ with respect to ${{\textbf {L}}}^{{-}}$. This leads to the following optimization problem:

3 $$\begin{aligned}\mathop {\textrm{minimize}}\limits _{{{\textbf {L}}}^{{+}} \in {\mathbb {L}}, {{\textbf{L}}}^{{-}} \in {\mathbb{L}}}\ \sum _{s \in \{+,-\}} \textrm{tr}({{\textbf{K}}}^{s} {\textbf {L}}^s) + \alpha _s \Vert {\textbf {L}}^s \Vert _{F}^2 \\\quad {{\,{\mathrm{subject\ to}}}}\ \ \textrm{tr}({\textbf {L}}^s) = 2n\ \forall s, \text { and } ({\textbf {L}}^+, {\textbf {L}}^-) \in {\mathbb {C}}, \end{aligned}$$

where ${\textbf {K}}^+ = {\textbf {X}}{\textbf {X}}^\top$, ${\textbf {K}}^- = -{\textbf {X}}{\textbf {X}}^\top$ and we used the cyclic property of trace operation, i.e. $\textrm{tr}({\textbf {X}}^\top {\textbf {L}}{\textbf {X}}) = \textrm{tr}({\textbf {X}}{\textbf {X}}^\top {\textbf {L}})$. ${{\textbf {L}}}^{{+}}$ and ${{\textbf {L}}}^{{-}}$ are constrained to be in the set $\mathbb {C}=\{({{\textbf {L}}}^{{+}}, {{\textbf {L}}}^{{-}}):L_{ij}^+=0 \text { if } L_{ij}^-\ne 0 \text { and } L_{ij}^-=0 \text { if } L_{ij}^+ \ne 0\}$ to ensure that they are not non-zero at the same indices.

The optimization problem in (3) can be kernelized to exploit various (nonlinear) relations between graph signals. Kernelization is important for GRN inference as it is unclear which association measure between gene expressions is best for various scRNA-seq data analysis [32]. Therefore, (3) is kernelized by changing ${\textbf {X}}{\textbf {X}}^\top$ with any positive semi-definite kernel matrix. In [31], three kernels are considered: correlation coefficient (*r*), proportionality measure ($\rho$) [34] and zero-inflated Kendall’s tau ($\tau _{zi}$) [35]. These kernels are considered because *r* is a commonly used metric for network inference, $\rho$ is found as the best performing measure in [32] and $\tau _{zi}$ can handle the dropouts in scRNA-seq.

### Multiview signed graph learning

Let $\{{\textbf {X}}^i\}_{i=1}^N$ be a given set consisting of *N* matrices. ${\textbf {X}}_i \in \mathbb {R}^{n \times p_i}$ is a data matrix constructed from $p_i$ graph signals defined on an unknown signed graph $G^i=(V, E^i, {\textbf {W}}^i)$ with $|V| = n$. It is assumed that $E^i$’s and associated edge weights are different but similar to each other. Based on this assumption, when learning $G^i$’s, one can have better performance by borrowing information across graphs. For example, when analyzing scRNA-seq expressions from different disease states/conditions, the datasets generated from the varying groups are generally assumed to share a common gene-coexpression structure. Thus, jointly learning cell-type specficic graphs can improve inference by allowing information sharing across cell-types. To this end, we propose an optimization problem (scMSGL) that learns $G^i$’s simultaneously. In the proposed approach, the learned $G^i$’s are regularized to be close to a consensus graph *G*, which is also learned by combining information from $G^i$’s. Thus, the proposed formulation ensures that information is shared across graphs when learning $G^i$’s. Furthermore, the structure of *G* reflects the common connections shared across $G^i$’s, whose inference may be beneficial if one is interested in learning the common gene-coexpression structure over the different cell-types/disease-stage subgroups.

Let ${\textbf {L}}^{i, +}$ and ${\textbf {L}}^{i, -}$ be the Laplacian matrices of the positive and negative parts of $G^i$, respectively. Similarly, define ${{\textbf {L}}}^{{+}}$ and ${{\textbf {L}}}^{{-}}$ for the consensus graph ${\textbf {G}}$. Let ${\mathcal {L}}^{{+}} = \{{\textbf {L}}^{1, +}, \dots , {\textbf {L}}^{N, +}, {\textbf {L}}^+\}$ and ${\mathcal {L}}^{{-}} = \{{\textbf {L}}^{1, -}, \dots , {\textbf {L}}^{N, -}, {\textbf {L}}^-\}$. The optimization problem for jointly learning $G^i$’s and *G* is then:

4 $$\begin{aligned}&\mathop {\textrm{minimize}}\limits _{{\mathcal {L}}^{{+}}, {\mathcal {L}}^{{-}}} \ \sum _{s\in \{+, -\}} \sum _{i=1}^N \big \{ \textrm{tr}({\textbf {K}}^{i,s} {\textbf {L}}^{i,s}) + \alpha _s \Vert {\textbf {L}}^{i,s} \Vert _F^2 + \beta _s \Vert {\textbf {L}}^{i,s} - {\textbf {L}}^s \Vert _{F,off}^2 \big \} \nonumber \\&\quad + \gamma _+ \Vert {\textbf {L}}^+ \Vert _{1,off} + \gamma _- \Vert {\textbf {L}}^- \Vert _{1,off} \nonumber \\ {{\,\mathrm{\mathrm{subject\ to}}\,}}\ {}&{\textbf {L}}^{i,s} \in \mathbb {L},\ \textrm{tr}({\textbf {L}}^{i, s}) = 2n,\ \forall i,\ \forall s \in \{+, -\}\ \nonumber \\&\quad ({\textbf {L}}^{i,+}, {\textbf {L}}^{i,-}) \in \mathbb {C}\ \forall i,{\textbf {L}}^+,\ {\textbf {L}}^- \in \mathbb {L},\ ({\textbf {L}}^{+}, {\textbf {L}}^{-}) \in \mathbb {C}, \end{aligned}$$

where ${\textbf {K}}^{i,+} = {\textbf {K}}^i$, ${\textbf {K}}^{i,-} = -{\textbf {K}}^i$, and ${\textbf {K}}^i$ is a kernel matrix constructed from ${\textbf {X}}^i$ as described in “Single view signed graph learning” section. $\Vert \cdot \Vert _{F,off}$ and $\Vert \cdot \Vert _{1,off}$ are the Frobenius norm and the $\varvec{\ell }_1$-norm of the off-diagonal entries, respectively. The first term in the summation measures the smoothness and non-smoothness of ${\textbf {X}}$ over $G^{i, +}$ and $G^{i, -}$, respectively. The second term controls the density of the learned $G^{i, +}$ ($G^{i, -}$) such that for larger values of $\alpha _+$ ($\alpha _-$), we learn denser graphs. The third term ensures that $G^{i, +}$ ($G^{i, -}$) are close to the positive (negative) part of consensus graph *G* with $\beta _+$ ($\beta _-$) controlling how close they should be. The last term is a regularizer that controls the sparsity of positive and negative parts of *G* with larger values of $\gamma _+$ and $\gamma _-$ resulting in a sparser consensus graph. Finally, the constraints are the same as in (3). Algorithm 1 gives the workflow of scMSGL to learn multiple graphs jointly. See Additional file 1 for an ADMM based optimization for (4).
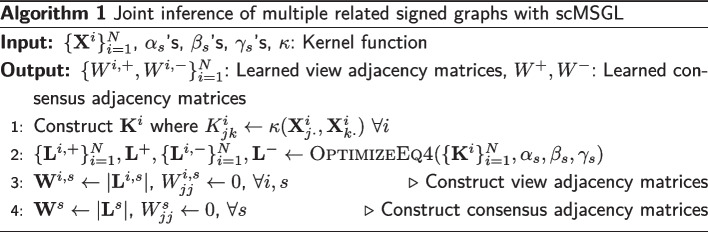


### Hyperparameter selection procedure

scMSGL requires the selection of six hyperparameters, three of which control the properties of the positive parts of the learned graphs while the remaining control the negative parts. As mentioned above, $\alpha _+$ ($\alpha _-$) and $\gamma _+$ ($\gamma _-$) control the edge density of positive (negative) parts of the learned $G^i$’s and *G*, respectively. $\beta _+$ ($\beta _-$) controls how similar the learned $G^{i, +}$’s ($G^{i, -}$’s) are to the consensus graph. We select these hyperparameters similar to that suggested in [6], where hyperparameter selection is guided to learn graphs with desired properties. Alternative to other model selection approaches, such as cross-validation or Bayesian information criterion, this approach can achieve a model that is interpretable and plausible in practice. Thus, we tune the hyperparameters such that the obtained graphs have a desired edge density and view similarity. In particular, assume that one wants the densities of positive and negative edges in the learned $G^i$’s and *G* to be $d_+$ and $d_-$, respectively. Furthermore, assume that the pairwise similarity between $G^{i,+}$ and $G^{j,+}$, $\forall i \ne j$ is desired to be $c_+$, where the similarity is quantified by the correlation coefficient. Similarly, let $c_-$ be the desired similarity amount for the negative edges of the graphs. Once $d_+$, $d_-$, $c_+$, $c_-$ are fixed, we select the six hyperparameters accordingly. The values of $d_+$, $d_-$, $c_+$, and $c_-$ are selected based on prior knowledge on the datasets under study as detailed in “Results” section.

## Results

The performance of scMSGL is evaluated on both simulated and two real scRNA-seq datasets. For simulated data, learned graphs are compared to ground truth networks to quantify the performance of scMSGL. Signed version of area under precision recall curve (AUPRC) is used as the performance metric during this analysis. We report AUPRC ratio, which is the ratio of AUPRC value of scMSGL to that of a random predictor. More details on how AUPRC is calculated can be found in Additional file 1. Simulated data are used to benchmark the performance of scMSGL against scSGL and three GRN inference algorithms, GENIE3, GRNBOOST2 and PIDC, which are found to be the best performing algorithms for scRNA-seq data [10]. These methods and scSGL can only learn a single graph from each dataset at a time. Therefore, they are applied to each ${\textbf {X}}^i$ separately and the learned graphs are compared to ground truth $G^i$’s. In addition, we benchmark against Joint Graphical Lasso with fused lasso penalty (JGL-Fused) method [6], which learns multiple related Gaussian graphical models, and Joint Gene Networks with scRNA-seq data (JGNsc) [21] algorithm, which jointly learns the graphs for multiple classes of single cell data. Other single cell joint graph learning algorithms discussed in “Background” section [17, 19] have not been considered due to the absence of publicly/on request available code.

### Selected hyperparameter values

Hyperparameters of scMSGL are set as described in “Methods” section with $d_+ = d_- = d$ and $c_+ = c_- = c$. We used the BEELINE [10] pipeline to run GENIE3, GRNBOOST2 and PIDC. GENIE3 and GRNBOOST2 employs random forest and gradient boosting regressors, respectively and hyperparameters of these regressors are set to the default values used in GENIE3 and GRNBOOST2 toolboxes. PIDC uses mutual information to learn gene regulations and it requires a discretizer and an estimator for probability distribution estimation. We used the discretizer and estimator recommended by PIDC toolbox. scSGL requires $\alpha _+$ and $\alpha _-$, which are determined the same way as $\alpha _s$’s of scMSGL, i.e., they are set to values such that learned graphs have desired edge densities of $d_+ = d_- = d$. JGL-Fused requires two parameters $\lambda _1$ and $\lambda _2$, which are analogous to the parameter of scMSGL, $\alpha _s$ and $\beta _s$, respectively. Therefore, they are set the same way, i.e. we choose $\lambda _1$ and $\lambda _2$ such that the learned graphs’ desired edge densities satisfy $d_+ + d_- = 2d$^1^ and view similarity of $c_+ = c_- = c$. Finally, JGNsc consists of three steps: imputation, Gaussian transformation and GRN inference with JFL-Fused method. The hyperparameters of the first two steps are set to the default values provided in JGNsc toolbox and $\lambda _1$ and $\lambda _2$ of JGL-Fused step are set as described above.^2^ Exact hyperparameter values of all methods are provided in Table 1 of Additional file 1.

For all datasets, we use $c=0.5$. For simulated data, since benchmarking GRN inference methods (GENIE3, GRNBOOST2 and PIDC) learn fully connected graphs, we set $d=0.4$ for fair comparison. For real data, we set $d=0.1$ for ease of analysis. See Additional file 1 for a discussion on the sensitivity of scMSGL to the selection of *d* and *c*.

### Simulated data

*Data generation:* To validate the performance of scMSGL, we simulate gene expression data from a multivariate zero-inflated negative binomial (ZINB) distribution. The ZINB distribution has been shown to accurately capture the characteristics of single cell datasets in several published studies [36, 37]. Given a known graph structure, we generate synthetic datasets using an algorithm developed by [38] and illustrated in [22, 31]. Two graph structures are considered for creating the baseline graph *G* with $n=100$ genes: random graphs following an Erdős-Rényi (ER) model with an edge density of 0.1 and hub graphs following a Barabási-Albert (BA) model with a degree distribution that follows the power-law. We then convert *G* to a signed graph by randomly selecting half of the edges and assigning a negative sign to them while assigning a positive sign to the other half. Next, we generate $N=5$ individual networks $\{G_i\}_{i=1}^{N}$ by adding $$0.9 \times \left( {\begin{array}{c}n\\ 2\end{array}}\right) \times \eta$$ new edges to the baseline graph *G*. Half of the added edges are set as negative edges, while the other half are set as positive. The ZINB simulator is then used to generate datasets $\{X_i\}_{i=1}^{N}$ from the underlying graphs $\{G_i\}_{i=1}^{N}$. The three parameters of the ZINB distribution; $\lambda$, *k* and $\omega$, which control its mean, dispersion and degree of zero-inflation, respectively were determined using a real scRNA-seq dataset [39]. Each simulation is repeated 10 times and the average performance over 10 realizations is reported. More details for data generation process can be found in Additional file 1.

**Fig. 1:**
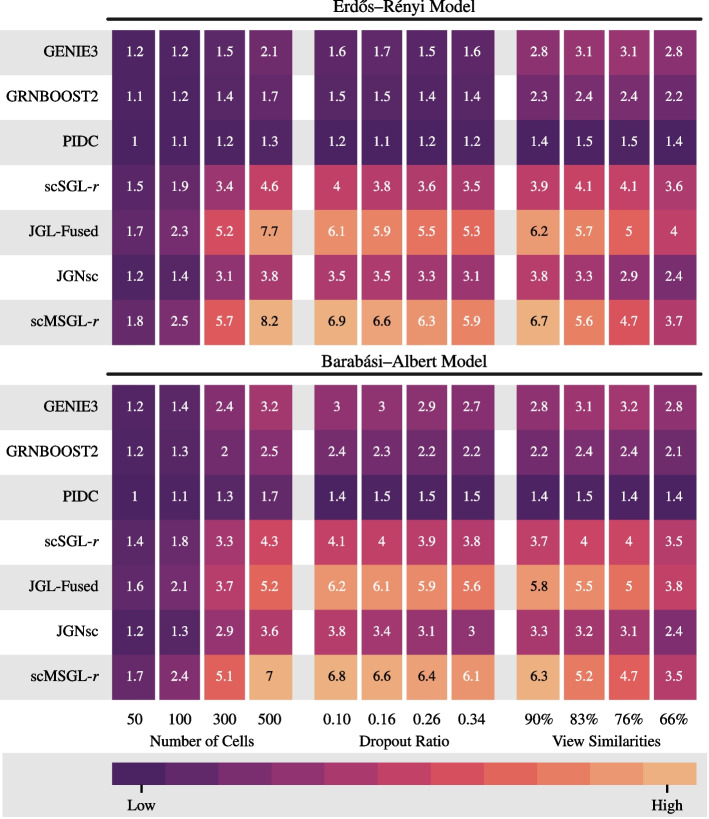
Performance of different methods on various datasets quantified by AUPRC ratio. All datasets have 100 genes. Left panel reports the results for varying number of cells. Middle one reports the results for varying dropout ratios. Right panel report results for varying degrees of view similarities, which is measured by the percentage of common edges across views in the ground truth graphs. Top plot shows the results for Erdős-Rényi model and the bottom plot shows the results for Barabási-Albert model

*Sensitivity to the number of cells:* We first study the performance of the methods with varying number of cells when the dropout ratio is set to 0.26, $\eta =0.1$, i.e. $90\%$ of the edges are common across views and the correlation kernel is used for both scSGL and scMSGL. From left panel of Fig. 1, it can be seen that for the different cell numbers, scMSGL has higher AUPRC ratios than methods that learn from a single dataset. This indicates that the proposed method incorporates valuable information across views, which improves the performance. scMSGL also performs better than both joint graph learning methods JGL-Fused and JGNsc. Although JGNsc is based on JGL-Fused, its performance is worse than JGL-Fused. This could be due to a change in the data structure owing to multiple imputation and data transformation steps, which form a part of the JGNsc algorithm. As expected, the performance of all methods improves with increasing number of cells. These observations hold for both random graph models.

*Sensitivity to dropout ratio:* In the second analysis, we evaluate the performance of the different methods with increasing dropout ratio while fixing the number of cells to 400 and $\eta =0.1$. Results are shown in the middle panel of Fig. 1 for both random graph models, with correlation kernel used for scSGL and scMSGL. Similar to cell sensitivity analysis, scMSGL performs better compared to all other methods irrespective of which graph model is used to generate the datasets. Except for PIDC, AUPRC ratios of all methods drop with increasing dropout ratio as expected. Performance of PIDC mostly remains the same. Since PIDC performs poorly at all drop-out levels, this result does not imply robustness against dropouts.

*Sensitivity to view similarity:* Next, we study the effect of view similarity on the performance of algorithms. Datasets are generated with varying $\eta$ values while fixing the number of cells to 400 and the dropout ratio to 0.26. Results are reported in right panel of Fig. 1, where the correlation kernel is employed for scSGL and scMSGL. When view similarity is 90%, the best performing algorithm is scMSGL, while for lower view similarity values JGL-Fused performs slightly better than scMSGL. The reason that JGL-Fused performs better than scMSGL for smaller view similarity values could be due to the difference in the regularization terms used to impose similarity across views. JGL-Fused uses a $\ell _1$ norm penalty, while we employ a squared Frobenius norm. Compared to fused lasso, squared Frobenius norm is susceptible to outliers, which can degrade the performance. The performance of single-view algorithms does not get affected by the changes in view similarity, as they learn each view independently. On the other hand, there is a drop in the performances of all joint graph learning methods with decreasing view similarity. This is an expected behaviour, since both methods assume the dependence of views.

**Fig. 2:**
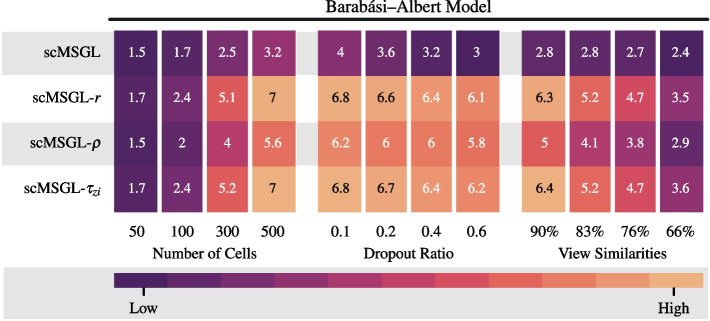
Performance of scMSGL without any kernel (first row) and with different kernels on datasets generated from BA model and studied in Fig. 1

*Kernel comparison:* Formulation of scMSGL allows us to use various kernels. Therefore, we study how the performance changes with respect to the kernel type. Datasets are created using the BA model and results are shown in Fig. 2 for varying number of cells, dropout ratios and view similarities. The best performing kernel is $\tau _{zi}$, followed by the correlation kernel. When Figs. 1 and 2 are compared, scMSGL has higher AUPRC ratios than single-view approaches and JGNsc irrespective of the kernel choice. The change in the performance of $\tau _{zi}$ and $\rho$ with varying cell numbers, dropout ratios and view similarity are very similar to that of the correlation. Finally, to better understand the effect of kernels, the performance of scMSGL without any kernels, i.e. ${\textbf {K}}^i = {\textbf {X}}^i{{\textbf {X}}^i}^\top$, is also reported. Figure 2 shows all kernels have significantly higher performance compared to when no kernel is used, which indicates the importance of kernel usage in GRN inference.

*Time complexity comparison:* We compare the different methods based on their run time complexity. We generated datasets using BA model with varying number of cells and number of genes. Table 1 reports the run time of scSGL, scMSGL, JGL-Fused and JGNsc in seconds. Run times of GENIE3, GRNBOOST2 and PIDC are not reported as they are shown to have higher time complexity than scSGL in [31]. Reported run times correspond to one run without hyperparameter search. Run time of scSGL is the total run time to infer all views.

In the first dataset, number of genes, dropout ratio and $\eta$ are fixed to 100, 0.26, and 0.1, respectively and number of cells varies. Results for this dataset indicate that scMSGL is faster than joint graph learning methods JGL-Fused and JGNsc. JGL-Fused also uses an ADMM based optimization, however it needs singular value decomposition at each ADMM iteration. scMSGL does not need this expensive operation; thus, it runs much faster than JGL-Fused. scSGL is faster than scMSGL, which is expected as scMSGL optimization takes longer time to converge due to added regularization terms and consensus graph learning. Finally, all methods except JGNsc are observed to run faster with increasing number of cells, since the inference problem becomes easier with higher number of cells, which makes iterative optimization procedure used by all methods converge faster. JGNsc runs slower with increasing number of cells, as its imputation step needs to handle a larger data matrix.

In the second dataset with increasing number of genes, the number of cells, dropout ration and $\eta$ are fixed to 500, 0.26, and $\eta =0.1$, respectively. As before, scMSGL is faster than joint graph learning methods and is slower than scSGL. Increasing the number of genes is observed to increase run time complexity of all methods, as it makes the problem harder.

**Table 1:** Run time of scMSGL and benchmarking methods in seconds with respect to number of cells and genes

	Number of cells				Number of genes			
Method	50	100	300	500	50	100	300	500
scSGL-*r*	1.10	0.54	0.35	0.38	0.15	0.37	5.88	26.68
JGL-Fused	175.64	117.65	95.95	98.03	10.02	95.98	1703.66	–
JGNsc	196.76	160.37	233.06	373.15	130.13	373.06	2541.45	–
scMSGL-*r*	14.00	12.39	10.00	8.51	0.35	3.89	110.49	304.71

All methods run on the same computing cluster with compute nodes that have similar compute power. Run times of JGL-Fused and JGNsc for 500 genes are not reported, we were not able to run them in a reasonable time limit (4 h)

### Analysis of scRNA-seq data from mouse embryonic stem cell differentiation

Central to the differentiation process and many other cellular processes is the expression of right combination of genes or modules of genes. Accurate characterization of the co-expression networks for progenitor and multiple cell types can help in understanding the cascade of cellular state transitions [12]. In this section, we study the differentiation process of mouse embryonic stem cells (mESC) using single cell RNA sequencing datasets [40]. This data was generated using high-throughput droplet-microfluidic approach and was primarily used to study differentiation in mESC before and after leukemia inhibitory factor (LIF) withdrawal. Since LIF maintains pluripotency of mESC, LIF withdrawal is considered to initiate the differentiation process. The dataset contains cells sampled from 4 states (or natural subgroups): before LIF withdrawal, day 0 and after the withdrawal for days 2, 4 and 7. The subgroups contain 933, 303, 683 and 798 cells, respectively. This dataset has been previously analyzed using joint graphical estimation in [17, 19] and similar to them we only consider the 72 stem cell markers in our application [41].^3^

**Fig. 3:**
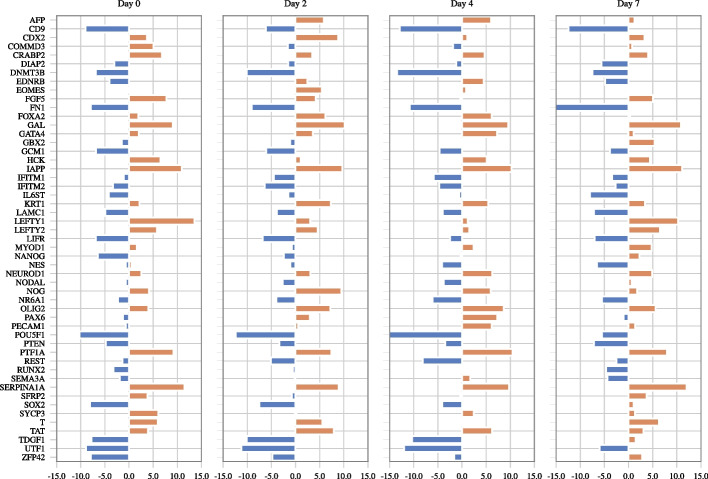
Genes with the highest node degrees. Orange and blue bars indicate that the degree is calculated using activating and inhibitory edges, respectively. Only genes whose activating or inhibitory degrees is among the top 15 genes in any view are shown

We first estimated the subgroup specific and the consensus graphs. Based on the results of simulated data, we employ the zero-inflated Kendall’s tau kernel. Next, we calculate the signed node degrees of each gene, i.e., $D_{ii}^+ = \sum _{j=1}^n W_{ij}^+$ and $D_{ii}^- = \sum _{j=1}^n W_{ij}^-$ from learned graphs $G^+$ and $G^-$. We then consider the genes with top signed degrees as hub genes whose signed degrees are reported in Fig. 3. The result confirms the importance of regulator genes NANOG, SOX2, POU5F1, ZFP42, UTF1 in early stages of differentiation. NANOG has been reported to maintain pluripotency by inhibiting genes that activate differentiation to lineages associated with extraembryonic endoderm [43, 44]. Figure  3 clearly shows that the number of inhibitory relationships associated with NANOG decreases as the ES cells proceed to a matured state. POU5F1 and SOX2 also exhibit higher number of inhibitory relationships in the the first few days. SOX2, NANOG and POU5F1 are known to play a fundamental role in the self-renewal and pluripotency of mouse embryonic stem cells [45]. Reduction in expression of NANOG has been shown to be correlated with the induction of gene GATA4 which initiates differentiation of pluripotent cells [46] and therefore GATA4 has been correctly identified as a hub gene in Days 2 and 4. Collectively, these results confirm the fundamental roles of SOX2, NANOG and POU5F1 in the pluripotency stage and how an eventual reduction in their expression initiates differentiation.

### Analysis of scRNA-seq data from medulloblastoma

Medulloblastoma (MB) is a highly malignant cerebellar tumor mostly affecting young children [47]. Several studies have been done to pinpoint the genetic drivers in each of the four distinct tumor subgroups: WNT-pathway-activated, SHH-pathway-activated, and the less-well-characterized Group 3 and Group 4 [47]. Among these subgroups, Group 3 and Group 4 tumors account for the majority of the MB diagnoses, with Group 3 MB having a metastatic diagnosis of approximately $50\%$. Transcription factors (TFs) MYC2 and OTX2 have commonly been identified as key oncogenic TFs in Group 3 and 4 tumorogenesis. Dong et al. [21] used the joint single cell network algorithm to study the roles of MYC and OTX2 utilizing the MB scRNA-seq data set (GSE119926) by [39].^4^ Using the same selected samples from a subset of 17 individuals that were grouped into three subsets Group 3, Group 4 and an intermediate cell type, we estimate the joint gene regulatory network for the three groups for $\sim 750$ genes among which most are enzyme-related genes from mammalian metabolic enzyme database [48]. Bulk profiling studies for MB cells have consistently observed overlapping transcriptional and epigenetic signatures in Group 3 and Group 4 tumors suggesting shared developmental origins [39, 49]. Based on this, we hypothesize that a joint analysis of the different MB cell-types would better capture the local functional interactions of MYC and OTX2 across different tumor subtypes and would eventually help in delineating their global role in regulating metabolic processes in MB cells.

**Fig. 4:**
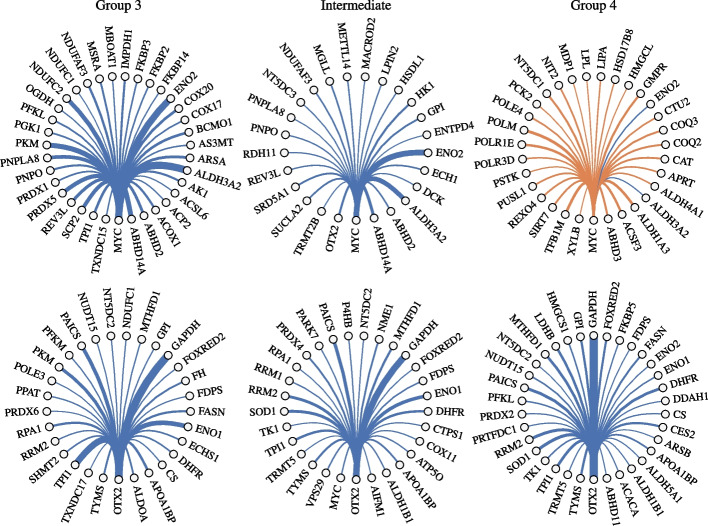
Connections of MYC (top) and OTX2 (bottom) genes. Edge widths are proportional to connection weights. Orange and blue edge colors indicate that the connection is activating and inhibitory, respectively. Only the top one third of the connections in all views of the multiview graph are shown

Subgroup specific networks along with the consensus graph were estimated with zero-inflated Kendall’s tau kernel. Table 2 shows that the average edge weight for the MYC network is considerably higher for Group 3 compared to Group 4 and the intermediate subgroup. Figure 4 further shows that Group 3 MYC network has stronger edge connections and higher density in compared to the intermediate group. In Group 4, almost all the connections become activating except for Aldh3a2 and Eno2; which were found to be strongly downregulated in all the tumor subgroups confirming their role in cancer resistance [50, 51]. This varying network structure over the subgroups confirms the major role MYC plays in initiation, maintenance, and progression of Group 3 tumors [52]. In Fig. 4, it is shown that OTX2 has a denser network for Group 4 MB cells in comparison to the other groups. In Group 3 MB cells, OTX2’s connections to the metabolic genes are very distinct from the MYC’s. In addition, scMSGL detected relationships between OTX2 and metabolic genes PAICS and PPAT in Group 3 tumors. These genes related to the human purine biosynthesis pathways have been previously reported to be induced by MYC [53]. This confirms that OTX2 is functionally cooperating with MYC to regulate gene expression in medulloblastoma [52, 54]. Broadly these results suggest that MYC and OTX2 play significant roles in in the transcriptional regulation of the metabolic genes and the mechanisms underlying MYC and OTX2 mediated MB maintenance and progression likely vary in different subgroups of MB cells.

**Table 2:** Node degree of MYC in learned graphs

	Group 3	Intermediate	Group 4
Total degree	5.436	3.334	4.180
Avg. edge weight	0.077	0.037	0.039

## Conclusion

In this paper, we presented scMSGL for joint inference of multiple GRNs from scRNA-seq datasets having multiple classes. scMSGL learns functional relationships between genes across multiple related classes of single cell gene expression datasets under the assumption that there exists a shared structure across classes. The main novelty of our paper lies in the formulation of a highly efficient optimization framework that extends the signed graph learning [31] approach to high dimensional datasets with multiple classes. The kernelization trick embedded within the algorithm renders it capable of handling sparse and noisy features; expected to demonstrate highly non-linear relationships. Furthermore, the estimation of the consensus graph may help in understanding the joint structure existing within the multiple classes. Using simulation studies, we demonstrated the superior performance of scMSGL over single view learning and existing joint learning methods for ER and BA graph models. In addition, performance was ascertained by varying a number of simulation parameters such as dropout levels, cell numbers and view similarity and scMSGL demonstrated superior performance in all scenarios. Applying scMSGL to the mESC dataset, we robustly identified previously reported regulatory markers as the hub genes for the different days and captured the progression of the differentiation process by analyzing these changes in hubs over the days. For the medulloblastoma data, scMSGL efficiently captured the significant roles that key oncology markers MYC and OTX2 play in the transcriptional regulation of metabolic genes.

There are various aspects of the proposed method that can be considered for improvement as future work. One challenge in implementing scMSGL is how to select the kernel function. This challenge can be addressed by combining information from multiple kernels during learning. An open problem in graph learning literature is hyperparameter selection, which is also a limitation of the proposed method. Current work selects the hyperparameters by searching the values that would result in graphs with desired properties. Future work can improve the accuracy of the learned graphs through better hyperparameter selection and multi-kernel strategies. Computational complexity of scMSGL is quadratic with respect to the number of genes (similar to scSGL) and linear in number of views. Therefore, its application to datasets with very large number of genes is not feasible. However, recent developments in GSP to scale GL to large-scale problems [55] can be exploited to scale scMSGL. Finally, additional sources of data that help in identifying direct interactions between TFs and target genes, can provide a way to filter out false positives. The current availability of single-cell epigenomic datasets has made it easier to further explore the regulatory relationship between TF and genes. Single-cell assay for transposase-accessible chromatin with sequencing (scATAC-seq), for example, allows the identification of DNA regulatory elements within accessible genomic DNA regions in single cells, hence enabling the identification of direct regulations in GRNs. Integration of multiomics profiles within the framework of scMSGL could be an interesting avenue for future research.

## Supplementary Information

**Additional file 1.** Additional file includes optimization process for scMSGL, definition of Signed AUPRC, simulated data generation process, results on sensitivity of scMSGL to hyperparameter selection and details about selected hyperparameters values of all methods.

## Data Availability

For real data, please see the cited references in “Results” section.The scMSGL code and simulated data are available at https://github.com/Single-Cell-Graph-Learning/scMSGL.

## Abbreviations

GRN: Gene regulatory networks
GSP: Graph signal processing
GL: Graph learning
GFT: Graph Fourier transform
scRNA-seq: Single cell RNA sequencing
scSGL: Single cell signed graph learning
scMSGL: Single cell multiple singed graph learning

## Footnotes

1 JGL-Fused does not allow edge densities of the negative and positive parts of the learned graph to be controlled separately, therefore we learned a graph with edge density equal to 2*d*, which is the same edge density for scMSGL if the edge signs are not considered.

2 JGNsc [21] recommends to use Akaike information criterion for selection of $\lambda _1$ and $\lambda _2$. In our analysis, we found this selection technique does not perform well and its time complexity was high.

3 The dataset was downloaded from GEO database [42] (with ID GSE65525). For the preprocessing steps, please refer to “Data analysis” subsection of the “Experimental procedures” section in [40]. The only preprocessing we performed was log-transformation to make count data continuous.

4 A detailed overview of the MB scRNA-seq data generation and processing can be found in the “Methods” section of [39] under subsection “Human scRNA-seq data generation and processing”. We log-transformed the obtained datasets to make count data continuous.
