# Supplementary material for: Kernelized multiview signed graph learning for single-cell RNA sequencing data
Source: BMC Bioinformatics. 2023 Apr 4;24:127. doi: 10.1186/s12859-023-05250-y (PMC10071725; doi:10.1186/s12859-023-05250-y)
Supplement: Supplementary file 1 — Additional file 1. Additional file includes optimization process for scMSGL, definition of Signed AUPRC, simulated data generation process, results on sensitivity of scMSGL to hyperparameter selection and details about selected hyperparameters values of all methods. [file 12859_2023_5250_MOESM1_ESM.pdf]

scMSGL: Additional File

Abdullah Karaaslanli[1], Satabdi Saha[2], Selin Aviyente[1] and Tapabrata Maiti[3]

[1]*Department of Electrical and Computer Engineering,*
Michigan State University, East Lansing, MI, USA

[2]*Department of Biostatistics,*
The University of Texas MD Anderson Cancer Center, Houston, TX, USA

[3]*Department of Statistics and Probability,*
Michigan State University, East Lansing, MI, USA

1. Optimization

In this section, an Alternating Direction Method of Multipliers (ADMM) based optimization procedure is described to solve scMSGL. For convenience, the problem formulation of scMSGL is given below:

$$\begin{aligned}
\underset{\mathcal{L}^+,\mathcal{L}^-}{\text{minimize}} \quad & \sum_{s\in\{+,-\}} \sum_{i=1}^{N} \left\{ \text{tr}(\mathbf{K}^{i,s}\mathbf{L}^{i,s}) + \alpha_s \|\mathbf{L}^{i,s}\|_F^2 + \beta_s \|\mathbf{L}^{i,s} - \mathbf{L}^s\|_{F,off}^2 \right\} + \gamma_+ \|\mathbf{L}^+\|_{1,off} + \gamma_- \|\mathbf{L}^-\|_{1,off} \\
\text{subject to } & \mathbf{L}^{i,s} \in \mathbb{L},\ \text{tr}(\mathbf{L}^{i,s}) = 2n,\ \forall i,\ \forall s \in \{+,-\}\ (\mathbf{L}^{i,+}, \mathbf{L}^{i,-}) \in \mathcal{C}\ \forall i, \\
& \mathbf{L}^+,\ \mathbf{L}^- \in \mathbb{L},\ (\mathbf{L}^+, \mathbf{L}^-) \in \mathcal{C}.
\end{aligned} \tag{1}$$

The problem in (1) can be written in a vectorized form, where one learns the upper triangular parts of the Laplacian matrices. For this, we first define some operators. $\text{diag}() : \mathbb{R}^{n\times n} \to \mathbb{R}^n$ is an operator that returns the diagonal of the input matrix. The operator $\text{upper}() : \mathbb{R}^{n\times n} \to \mathbb{R}^M$ returns the upper triangular part of the input matrix where $M = n(n-1)/2$. For an $n \times n$ symmetric matrix $\mathbf{A}$, we define the matrix $\mathbf{S} \in \mathbb{R}^{M\times n}$ such that $\mathbf{S}\text{upper}(\mathbf{A}) = \mathbf{A}\mathbf{1} - \text{diag}(\mathbf{A})$. Finally, let $\mathbf{1}$ and $\mathbf{0}$ be all-one and all-zero vectors. To vectorize (1), let $\mathbf{k}^{i,s} = \text{upper}(\mathbf{K}^{i,s})$, $\mathbf{d}^i = \text{diag}(\mathbf{K}^{i,s})$, $\boldsymbol{\ell}^{i,s} = \text{upper}(\mathbf{L}^{i,s})$ and $\boldsymbol{\ell}^s = \text{upper}(\mathbf{L}^s)$ for $s \in \{+,-\}$. Also, let $\mathcal{L}_v^+ = \{\boldsymbol{\ell}^{1,+}, \dots, \boldsymbol{\ell}^{N,+}, \boldsymbol{\ell}^+\}$ and $\mathcal{L}_v^- = \{\boldsymbol{\ell}^{1,-}, \dots, \boldsymbol{\ell}^{N,-}, \boldsymbol{\ell}^-\}$. The vectorized form of (1) is:

$$\begin{aligned}
\underset{\mathcal{L}_v^+,\mathcal{L}_v^-}{\text{minimize}} \quad & \sum_{s\in\{+,-\}} \sum_{i=1}^{N} \left\{ \langle \mathbf{k}^{i,s} - \mathbf{S}^\top \mathbf{d}^{i,s}, \boldsymbol{\ell}^{i,s} \rangle + \alpha \|-\mathbf{S}\boldsymbol{\ell}^{i,s}\|_2^2 + 2\alpha \|\boldsymbol{\ell}^{i,s}\|_2^2 + \beta \|\boldsymbol{\ell}^{i,s} - \boldsymbol{\ell}^s\|_2^2 \right\} + \gamma_+ \|\boldsymbol{\ell}^+\|_1 - \gamma_- \|\boldsymbol{\ell}^-\|_1 \\
\text{subject to } & \mathbf{1}^\top \boldsymbol{\ell}^{i,+} = -n, \mathbf{1}^\top \boldsymbol{\ell}^{i,-} = -n, \boldsymbol{\ell}^{i,+} \leqslant 0,\ \boldsymbol{\ell}^{i,-} \leqslant 0,\ \boldsymbol{\ell}^{i,+} \perp \boldsymbol{\ell}^{i,-}\ \forall i \text{ and } \boldsymbol{\ell}^+ \leqslant 0,\ \boldsymbol{\ell}^- \leqslant 0,\ \boldsymbol{\ell}^+ \perp \boldsymbol{\ell}^-,
\end{aligned} \tag{2}$$

where the first term in the summation corresponds to the first term in (1), and the correspondence between the remaining terms to the term in (1) can be deduced using the hyperparameters. First two constraints correspond to the trace constraints in (1). The constraint $\boldsymbol{\ell}^{i,+} \perp \boldsymbol{\ell}^{i,-}$ together with $\boldsymbol{\ell}^{i,+} \leqslant 0$ and $\boldsymbol{\ell}^{i,-} \leqslant 0$ is called complementarity constraints (Scheel and Scholtes, 2000) and corresponds to $(\mathbf{L}^{i,+}, \mathbf{L}^{i,-}) \in \mathcal{C}$ in (1).

The problem in (2) is non-convex due to complementarity constraints. However, ADMM is shown to

be convergent for problems with complementarity constraints under some assumptions (Wang *et al.*, 2019). To write the problem in standard ADMM form, introduce auxiliary variables $\mathbf{v}^i = \boldsymbol{\ell}^{i,+}$ and $\mathbf{w}^i = \boldsymbol{\ell}^{i,-}$ for all i. Similarly, introduce $\mathbf{v} = \boldsymbol{\ell}^+$ and $\mathbf{w} = \boldsymbol{\ell}^-$. Also, let $\mathcal{V} = \{\mathbf{v}^1, \ldots, \mathbf{v}^N, \mathbf{v}\}$ and $\mathcal{W} = \{\mathbf{w}^1, \ldots, \mathbf{w}^N, \mathbf{w}\}$. Then, the problem in its standard ADMM form is:

$$\begin{aligned} &\underset{\mathcal{L}_v^+,\mathcal{L}_v^-,\mathcal{V},\mathcal{W}}{\text{minimize}} \sum_{i=1}^N \iota_S(\mathbf{v}^i,\mathbf{w}^i) + \sum_{s\in\{+,-\}}\sum_{i=1}^N \left\{ f(\boldsymbol{\ell}^{i,s},\boldsymbol{\ell}^s) + \iota_H(\boldsymbol{\ell}^{i,s}) \right\} + \iota_S(\mathbf{v},\mathbf{w}) + \gamma_+\|\boldsymbol{\ell}^+\|_1 - \gamma_-\|\boldsymbol{\ell}^-\|_1 \\ &\text{subject to} \quad \mathbf{v}^i = \boldsymbol{\ell}^{i,+},\ \mathbf{w}^i = \boldsymbol{\ell}^{i,-},\ \mathbf{v} = \boldsymbol{\ell}^+,\ \text{and } \mathbf{w} = \boldsymbol{\ell}^-, \end{aligned} \tag{3}$$

where $f(\boldsymbol{\ell}^{i,s},\boldsymbol{\ell}^s) = \langle \mathbf{k}^{i,s} - \mathbf{S}^\top\mathbf{d}^{i,s}, \boldsymbol{\ell}^{i,s}\rangle + \alpha\|-\mathbf{S}\boldsymbol{\ell}^{i,s}\|_2^2 + 2\alpha\|\boldsymbol{\ell}^{i,s}\|_2^2 + \beta\|\boldsymbol{\ell}^{i,s} - \boldsymbol{\ell}^s\|_2^2$, $\iota_S(\cdot,\cdot)$ is the indicator function for the complementarity set $S = \{(\mathbf{v},\mathbf{w}) : \mathbf{v} \leqslant 0,\ \mathbf{w} \leqslant 0,\ \mathbf{v} \perp \mathbf{w}\}$, and $\iota_H(\cdot)$ is the indicator function for the hyperplane $H = \{\boldsymbol{\ell} : \mathbf{1}^\top\boldsymbol{\ell} = -n\}$. Augmented Lagrangian can then be written as:

$$\begin{aligned} L_p(\mathcal{L}_v^+,\mathcal{L}_v^-,\mathcal{V},\mathcal{W}) = &\sum_{i=1}^N \iota_S(\mathbf{v}^i,\mathbf{w}^i) + \sum_{s\in\{+,-\}}\sum_{i=1}^N \left\{ f(\boldsymbol{\ell}^{i,s},\boldsymbol{\ell}^s) + \iota_H(\boldsymbol{\ell}^{i,s}) \right\} \\ &+ \sum_{i=1}^N \left\{ \lambda_{i,+}^\top(\mathbf{v}^i - \boldsymbol{\ell}^{i,+}) + \frac{\rho}{2}\|\mathbf{v}^i - \boldsymbol{\ell}^{i,+}\|_2^2 + \lambda_{i,-}^\top(\mathbf{w}^i - \boldsymbol{\ell}^{i,-}) + \frac{\rho}{2}\|\mathbf{w}^i - \boldsymbol{\ell}^{i,-}\|_2^2 \right\} \\ &+ \iota_S(\mathbf{v},\mathbf{w}) + \gamma_+\|\boldsymbol{\ell}^+\|_1 - \gamma_-\|\boldsymbol{\ell}^-\|_1 \\ &+ \lambda_+^\top(\mathbf{v} - \boldsymbol{\ell}^+) + \frac{\rho}{2}\|\mathbf{v} - \boldsymbol{\ell}^+\|_2^2 + \lambda_+^\top(\mathbf{w} - \boldsymbol{\ell}^-) + \frac{\rho}{2}\|\mathbf{w} - \boldsymbol{\ell}^-\|_2^2, \end{aligned} \tag{4}$$

where ρ is the parameter of augmented Lagrangian, $\lambda_{i,+}$, $\lambda_{i,-}$, λ_+ and λ_- are the Lagrange multipliers. Using augmented Lagrangian, ADMM steps at kth iteration are then found as follows:

$$(\widehat{\mathcal{V}},\widehat{\mathcal{W}}) = \underset{\mathcal{V},\mathcal{W}}{\operatorname{argmin}}\, L_p(\widehat{\widehat{\mathcal{L}}}_v^+,\widehat{\widehat{\mathcal{L}}}_v^-,\mathcal{V},\mathcal{W}), \tag{5}$$

$$(\widehat{\mathcal{L}}_v^+,\widehat{\mathcal{L}}_v^+) = \underset{\mathcal{L}_v^+,\mathcal{L}_v^-}{\operatorname{argmin}}\, L_p(\mathcal{L}_v^+,\mathcal{L}_v^-,\widehat{\mathcal{V}},\widehat{\mathcal{W}}), \tag{6}$$

$$\widehat{\lambda}_{i,+} = \widehat{\widehat{\lambda}}_{i,+} + \rho(\widehat{\mathbf{v}}^i - \widehat{\boldsymbol{\ell}}^{i,+}),\ \forall i, \tag{7}$$

$$\widehat{\lambda}_{i,-} = \widehat{\widehat{\lambda}}_{i,-} + \rho(\widehat{\mathbf{w}}^i - \widehat{\boldsymbol{\ell}}^{i,-}),\ \forall i, \tag{8}$$

$$\widehat{\lambda}_+ = \widehat{\widehat{\lambda}}_+ + \rho(\widehat{\mathbf{v}} - \widehat{\boldsymbol{\ell}}^+), \tag{9}$$

$$\widehat{\lambda}_- = \widehat{\widehat{\lambda}}_- + \rho(\widehat{\mathbf{w}} - \widehat{\boldsymbol{\ell}}^+), \tag{10}$$

where $\widehat{}$ and $\widehat{\widehat{}}$ represent the values of variables at kth and $(k-1)$th iteration, respectively. To solve (5), we use the fact that it can be solved for each ($\mathbf{v}^i$, $\mathbf{w}^i$) pair (and ($\mathbf{v}$, $\mathbf{w}$)), separately. This separation leads to a set of optimization problems all of which can be solved by projection onto the complementarity set S. The problem in (6) is separable across $\mathcal{L}_v^+$ and $\mathcal{L}_v^-$, leading to two optimization problems both of which can be solved with Block Coordinate Descent (BCD) (Shi *et al.*, 2016).

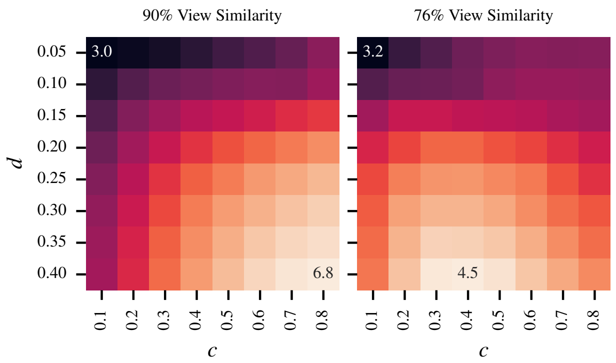

Figure 1: Sensitivity of scMSGL to hyperparameter selection. Squares with values indicate the lowest and highest AUPRC ratios.

2. Signed AUPRC Ratio

Edges in GRNs can be either activating, inhibitory or non-existing. Thus, performance of a GRN inference algorithm should be measured by how well it can infer activating, inhibitory and non-existing edges. Given the ground truth GRN G and the output of a GRN inference algorithm $\widehat{G}$, let G^+ and G^- be the activating and inhibitory edges in the ground truth GRN and $\widehat{G}^+$ and $\widehat{G}^-$ be the activating and inhibitory edges in the inferred network. We compare $\widehat{G}^+$ to G^+ with AUPRC to measure how well the algorithm finds the activating edges. Similarly, we compare $\widehat{G}^-$ to G^- to measure the performance on inhibitory edges. Let $AUPRC^+$ and $AUPRC^-$ represent these values. We calculate signed AUPRC ratio as follows:

$$SignedAUPRCRatio = \frac{1}{2}\left(\frac{AUPRC^+}{AUPRC^+_{random}} + \frac{AUPRC^-}{AUPRC^-_{random}}\right), \tag{11}$$

where $AUPRC^+_{random}$ and $AUPRC^-_{random}$ are the performance measures of a random estimator. Finally, note that if an algorithm infers an unsigned GRN, we use the inferred GRN for both $\widehat{G}^+$ and $\widehat{G}^-$.

3. Hyperparameter Sensitivity

In this section, we study the sensitivity of scMSGL to hyperparameter selection. As described above, hyperparameters are selected by setting d_+, d_-, c_+ and c_-. We set $d_+ = d_- = d$ and $c_+ = c_- = c$ and change the values of c and d. In Figure 1, we report the performance of scMSGL with correlation kernel for varying c and d values. The two datasets from the right panel of Figure 1 of the main text are considered. In both datasets, the highest AUPRC ratios are obtained when the learned graphs are the densest. Comparing left and right plots of the Figure 1, it is also observed that the optimal value of c changes with decreasing view similarity in the ground truth graphs. Note that the lowest AUPRC ratio is still comparable to or higher than those of benchmarking algorithms reported in Figure 1 of the main text. Overall, it can be concluded that there is a large range of values around the optimal d and c for which the learned graphs have satisfactory AUPRC ratios.

4. Data Generation Procedure

1. For each simulation setting, we first draw a baseline binary graph G from a random graph model with n genes. We considered Erdős–Rényi and Barabasi-Albert random graph models as mentioned in the main text.

2. $N = 5$ perturbed graphs are generated by randomly adding $0.9 \times \binom{n}{2} \times \eta$ edges to the baseline graph where $\eta > 0$ is the fraction of added edges.

3. For the kth graph, each edge is assigned a weight, W_{ij}^k such that:

$$W_{ij}^k = \begin{cases} \mathtt{Unif}(0.3, 0.7) & \text{with probability } 0.5, \\ \mathtt{Unif}(-0.7, -0.3) & \text{otherwise.} \end{cases}$$

4. For the kth graph, p_k random samples are drawn from multivariate Gaussian distribution with precision matrix $\mathbf{W}^k$. The random samples are used as the columns of the matrix $\mathbf{X}^k \in \mathbb{R}^{n \times p_k}$.

5. To mimic the dropout phenomenon present in real single cell datasets, we next introduced additional zeros to the gene expression matrix $\mathbf{X}^k$. Following (Pierson and Yau, 2015), the dropout probability for each row of $\mathbf{X}^k$ was calculated as: $\pi_{ij} = \exp(-\alpha {X_{ij}^k}^2)$, where α represents the exponential decay parameter that controls the dependence between the dropout probability and gene expression.

6. A binary indicator was next sampled for each entry: $\xi_{ij}^k \sim \mathtt{Bernoulli}(\pi_{ij})$, with $\xi_{ij}^k = 1$ indicating that the corresponding entry of X_{ij}^k would be replaced by 0. The dropout probability for each gene vector was calculated as $\omega_i^k = \sum_{j=1}^{p_k} \xi_{ij}$.

7. Using a modification of the NORTA (Normal to Anything) method (Yahav and Shmueli, 2012) we generated samples from a multivariate zero inflated negative binomial distribution based on $\mathbf{X}^k$ generated in Step 4 using mean λ, dispersion κ and zero-inflation parameters ω_j's.

8. To mirror real scRNA-seq gene expression data behaviour, λ and κ were estimated from the MB scRNA-seq data set (GSE119926) (Hovestadt *et al.*, 2019) analyzed in the main manuscript.

References

Hovestadt, V. *et al.* (2019). Resolving medulloblastoma cellular architecture by single-cell genomics. *Nature*, **572**(7767), 74–79.

Pierson, E. and Yau, C. (2015). Zifa: Dimensionality reduction for zero-inflated single-cell gene expression analysis. *Genome biology*, **16**(1), 1–10.

Scheel, H. and Scholtes, S. (2000). Mathematical programs with complementarity constraints: Stationarity, optimality, and sensitivity. *Mathematics of Operations Research*, **25**(1), 1–22.

Shi, H.-J. M. *et al.* (2016). A primer on coordinate descent algorithms. *arXiv preprint arXiv:1610.00040*.

Wang, Y. *et al.* (2019). Global convergence of admm in nonconvex nonsmooth optimization. *Journal of Scientific Computing*, **78**(1), 29–63.

Yahav, I. and Shmueli, G. (2012). On generating multivariate poisson data in management science applications. *Applied Stochastic Models in Business and Industry*, **28**(1), 91–102.

Method	Hyperparameters
GENIE3	*Max Features*: $\sqrt{n}$<br>*Number of Estimators*: 1000
GRNBOOST2	*Learning Rate*: 0.01,<br>*Number of Estimators*: 5000<br>*Subsample*: 0.9
PIDC	*Discretizer*: Bayesian blocks algorithm<br>*Estimator*: Maximum likelihood
scSGL	α_+: Search for the value that returns graphs with $d_+ = d$<br>α_-: Search for the value that returns graphs with $d_- = d$
scMSGL	α_+ and γ_+: Search for the value that returns graphs with $d_+ = d$<br>α_- and γ_-: Search for the value that returns graphs with $d_- = d$<br>β_+: Search for the value that returns graphs with $c_+ = c$<br>β_-: Search for the value that returns graphs with $c_- = c$
JGL-Fused	λ_1: Search for the value that returns graphs with edge density 2d<br>λ_2: Search for the value that returns graphs with $c_+ = c_- = c$
JGNsc	*Masking Rate*: 0.15<br>*Number of Iterations in the Imputation Procedure*: 50<br>*MCMC Warm Steps*: 1000<br>*MCMC Iterations*: 5000<br>α: 3, β: 1<br>*Dropout Threshold*: 0.75<br>λ_1: Search for the value that returns graphs with edge density 2d<br>λ_2: Search for the value that returns graphs with $c_+ = c_- = c$

Table 1: Selected hyperparameters of baseline methods and scMSGL. n is the number of genes, d_+ and d_- are the desired positive and negative edge densities of the learned graphs, respectively. c_s is the desired pairwise similarity between $G^{i,s}$ and $G^{j,s}$, $\forall i \neq j$ and $s \in [+,-]$.
